# ADAM-17 is expressed on rheumatoid arthritis fibroblast-like synoviocytes and regulates proinflammatory mediator expression and monocyte adhesion

**DOI:** 10.1186/s13075-018-1657-1

**Published:** 2018-08-02

**Authors:** Sho Ishii, Takeo Isozaki, Hidekazu Furuya, Hiroko Takeuchi, Yumi Tsubokura, Katsunori Inagaki, Tsuyoshi Kasama

**Affiliations:** 1 Division of Rheumatology, Department of Medicine, Showa University School of Medicine, 1-5-8 Hatanodai, Shinagawa-ku, Tokyo, 142-8666 Japan; 2 Department of Orthopedic Surgery, Showa University School of Medicine, Tokyo, Japan

**Keywords:** ADAM-17, Rheumatoid arthritis, Cell adhesion, Cytokines

## Abstract

**Background:**

To examine the expression of ADAM-17 in rheumatoid arthritis (RA) biological fluids and the role it plays in monocyte adhesion to RA fibroblast-like synoviocytes (FLSs).

**Methods:**

ADAM-17 expression was measured by enzyme-linked immunosorbent assays (ELISAs) in serum from normal (NL) subjects, osteoarthritis (OA) patients, and RA patients. We also analyzed the correlation between ADAM-17 and disease activity score 28 (DAS28) in RA. To determine expression of ADAM-17 in RA synovial tissues (STs) and RA FLS, we performed immunofluorescence analyses. To determine the role of ADAM-17 in RA, we transfected RA FLSs with small interfering RNA (siRNA) against ADAM-17. THP-1 adhesion to ADAM-17 siRNA-transfected RA FLSs was measured. Finally, adhesion molecules on ADAM-17 siRNA-transfected RA FLSs were measured using cell surface ELISAs.

**Results:**

ADAM-17 in RA serum was significantly higher than that in NL and OA serum and correlated with DAS28. ADAM-17 in RA synovial fluids was higher than that in OA synovial fluids. ADAM-17 was expressed on RA cells lining STs and RA FLSs. THP-1 adhesion to ADAM-17 siRNA-transfected RA FLSs was decreased compared with that to control siRNA-transfected RA FLSs. ICAM-1 on TNF-α-stimulated ADAM-17 siRNA-transfected RA FLSs was significantly decreased compared with that on control siRNA-transfected RA FLSs.

**Conclusions:**

These data indicate that ADAM-17 is expressed on RA STs and plays a role in RA inflammation by regulating monocyte adhesion to RA FLSs. ADAM-17 might be an important inflammatory mediator in inflammatory diseases such as RA.

## Background

Rheumatoid arthritis (RA) is a systemic disorder and common autoimmune disease characterized by chronic synovial inflammation that leads to joint destruction and bone erosion [1]. Although the pathogenesis of RA is only partially understood, inflammatory cells, macrophages, lymphocytes, and fibroblasts are known to produce several cytokines and chemokines in RA inflammation [2]. Determining the cytokine and chemokine networks in RA will help elucidate the pathogenesis of RA and can contribute to development of treatments [3]. In recent decades, RA therapies have changed and have shown improved outcomes. One of the most effective current therapies is biologic disease-modifying therapies, which are designed to block tumor necrosis factor α (TNF-α) and interleukin (IL) -6 [4, 5]. Anti-CD20 therapies which deplete B-cell and T-cell regulation therapies by binding cytotoxic lymphocyte antigen-4 are also highly effective treatments for RA [6, 7]. Although these therapies have been shown to be effective in RA patients, they sometimes fail or produce only partial responses [8–10]. Currently, studies using antibodies to other chemokines or chemokine inhibitors have been conducted in animal models of arthritis [11]. Discovering specific chemokine or chemokine receptor targets may contribute to the development of RA treatment with other molecules.

A disintegrin and metalloproteases (ADAMs) are a family of surface-expressed and secreted proteins that contain metalloproteinase domains [12]. In this family, ADAM-17 is known as a TNF-α-converting enzyme, which is the principal protease involved in the activation of pro-TNF-α. ADAM-17 also has shedding functions and plays a role in a broad range of cell surface molecules. In particular, ADAM-17 has been shown to be involved in generation of the active forms of epidermal growth factor receptor (EGFR) ligands; cytokine receptors, such as IL-6 receptors, and TNF receptors; and adhesion proteins, such as intercellular adhesion molecule (ICAM)-1 [13–15]. Through these molecules, ADAM-17 affects immune and inflammatory responses and cancer development [16]. In addition, ADAM-17 has been related to various diseases, including lung cancer, polycystic kidney, Alzheimer’s disease, and autoimmune diseases, such as RA [17–20]. However, the role of ADAM-17 in RA inflammation is still unclear. In this study, we showed that ADAM-17 is expressed on RA synovial tissues (STs) and fibroblast-like synoviocytes (FLSs) and mediates monocyte adhesion and production of proinflammatory cytokines in RA.

## Methods

### Patients

RA STs were obtained from patients undergoing arthroplasty or synovectomy. RA and osteoarthritis (OA) synovial fluids (SFs) were obtained from patients. RA, OA, and normal (NL) serum were collected. All specimens were obtained with informed consent and collected following approval from the Showa University Institutional Review Board.

### Cell culture

Fresh STs were minced and digested in tissue enzyme digestion solution as described previously [21]. FLSs were maintained in RPMI 1640 medium supplemented with 10% fetal bovine serum (FBS). Cells were seeded in six-well plates (BD Biosciences, Bedford, MA, USA) at a density of 1 × 10^5^ cells per well and were maintained in complete medium. After overnight serum starvation, the cells were treated with TNF-α (R&D Systems, Minneapolis, MN, USA).

THP-1 cells (a human acute monocytic leukemia cell line) were purchased from the American Type Culture Collection (Manassas, VA, USA). THP-1 cells were cultured in RPMI 1640 medium supplemented with 10% FBS.

### Enzyme-linked immunosorbent assays (ELISAs) of ADAM-17, fractalkine/CX3CL1, and CXCL16

ELISAs were performed as described previously [22]. The ADAM-17 levels in serum and SFs were measured following the manufacturer’s protocol (R&D Systems). Briefly, 96-well plates were coated with mouse anti-ADAM-17 antibody. Next day, RA, NL serum or recombinant ADAM-17, which was used as a standard biotinylated anti-human ADAM-17 antibody (R&D Systems) was added as a detection antibody, followed by streptavidin-HRP (BD Biosciences). The plates were developed using tetramethylbenzidine substrate (TMB, Sigma-Aldrich, St Louis, MO, USA) and were read on a microplate reader at 450 nm.

Fractalkine/CX3CL1 and CXCL16 in RA FLS-conditioned medium were measured using an R&D Duo kit (R&D Systems). ADAM-17 or control siRNA-transfected RA FLS were stimulated with TNF-α (10 ng/ml) for 24 h.

### Immunofluorescence

For analysis of ADAM-17 expression in RA STs and RA FLSs, rabbit anti-ADAM-17 was used as the primary antibody. (High-activity STs were selected from RA STs using hematoxylin and eosin stain.) (To measure the secretion of ADAM-17 in RA ST synovial fibroblasts, mouse anti-human collagen-1 and rabbit anti-ADAM-17 were used as primary antibodies.) RA FLSs were plated at a density of 20,000/well in eight-well Labtek chamber slides. The next day, the cells were washed with PBS and fixed. RA ST slides were fixed with cold acetone for 20 min and washed with PBS. Then, the slides were blocked with 20% FBS and 5% donkey serum for 1 h at 37 °C. Rabbit anti-human ADAM-17 antibody (10 μg/ml, Abcam, Cambridge, MA, USA) and mouse anti-human collagen-1 antibody (1 μg/ml, Abcam) was used. Alexa Fluor 488-conjugated donkey anti-rabbit antibody and Alexa Flour 555-conjugated donkey anti-mouse antibody were purchased from Life Technologies (Carlsbad, CA, USA). For nuclear staining, 4′,6-diamidino-2-phenylindole (DAPI) was used. Images were taken at ×200 magnification.

### RNA isolation and quantitative PCR (qPCR)

RA FLS were incubated in six-well plates in 0.1% BSA RPMI medium overnight, before stimulation with TNF-α (R&D Systems, 10 ng/ml). Total RNA was isolated from RA FLS using RNAeasy mini RNA isolation kits in conjunction with QIAshredders (Qiagen, Valencia, CA, USA) following the manufacturer’s protocol. Following isolation, RNA was quantified and checked for purity using a spectrophotometer (Nanodrop Technologies, Wilmington, DE, USA). cDNA was then prepared using a Verso cDNA kit (Thermo Fisher Scientific, Waltham, MA, USA) as per the manufacturer’s protocol. Quantitative PCR (qPCR) was performed using Platinum SYBR Green qPCR SuperMix-UDG (Invitrogen, Carlsbad, CA, USA) following the manufacturer’s protocol. All samples were run in duplicate and using Eppendorf software.

### Transfection of RA FLSs with ADAM-17 small interfering RNA (siRNA)

RA FLSs were seeded in six-well plates at a density of 1 × 10^5^ cells per well. siRNA (100 nM) against ADAM-17 or control siRNA was mixed with TransIT-TKO transfection reagent (Mirus, Madison, WI, USA) according to the manufacturer’s instructions and overlaid on the cells. The cells were incubated with the siRNA/TransIT-TKO for 24 h at 37 °C. The ADAM-17 and control siRNAs were purchased from Santa Cruz Biotechnology (Dallas, TX, USA). Knockdown of ADAM-17 secretion was confirmed using Western blotting. Western blotting was performed as described previously [23]. Membranes were probed with rabbit anti-human ADAM-17 antibody (Abcam, Cambridge, MA, USA) and anti-βactin.

### In vitro cell adhesion assay

The adhesion of THP-1 cells to control siRNA-treated or ADAM-17 siRNA-treated RA FLSs grown to confluence in 96-well plates was examined. RA FLSs were serum-starved overnight. The next day, the cells were treated with TNF-α (10 ng/ml) for 24 h. THP-1 cells were collected and labeled with calcein AM fluorescent dye (Life Technologies, 5 μM) for 30 min. After being washed twice, 1 × 10^5^ THP-1 cells were added to each well and incubated for 30 min at room temperature. Nonadherent cells were washed away, and the fluorescence was measured using a Synergy HT fluorescence plate reader (BioTek Instruments, Winooski, VT, USA).

### Cell surface ELISA for adhesion molecule expression

Control siRNA-transfected or ADAM-17 siRNA-transfected RA FLSs (1 × 10^5^/well) were seeded in 96-well plates. Confluent RA FLSs were serum-starved overnight prior to stimulation with TNF-α (10 ng/ml) for 24 h. The cells were fixed with 3.7% formalin in PBS for 30 min. Mouse anti-human antibodies specific for intercellular adhesion molecule 1 (ICAM-1, 10 μg/ml, R&D Systems) or vascular cell adhesion molecule 1 (VCAM-1) were incubated for 1 h. Subsequently, biotinylated anti-mouse antibody and streptavidin-HRP were added for 1 h, and the concentration of the samples was measured at 450 nm after reaction with the tetramethylbenzidine substrate.

### Statistical analysis

The data were analyzed using Student’s *t* test assuming equal variances. The relationship between ADAM-17 in RA serum and a disease activity score of 28 (DAS28) was evaluated using Spearman’s rank correlation. Data are reported as the mean ± SEM. *P* values less than 0.05 were considered statistically significant.

## Results

### Expression of ADAM-17 in RA serum and SFs

**Fig. 1:**
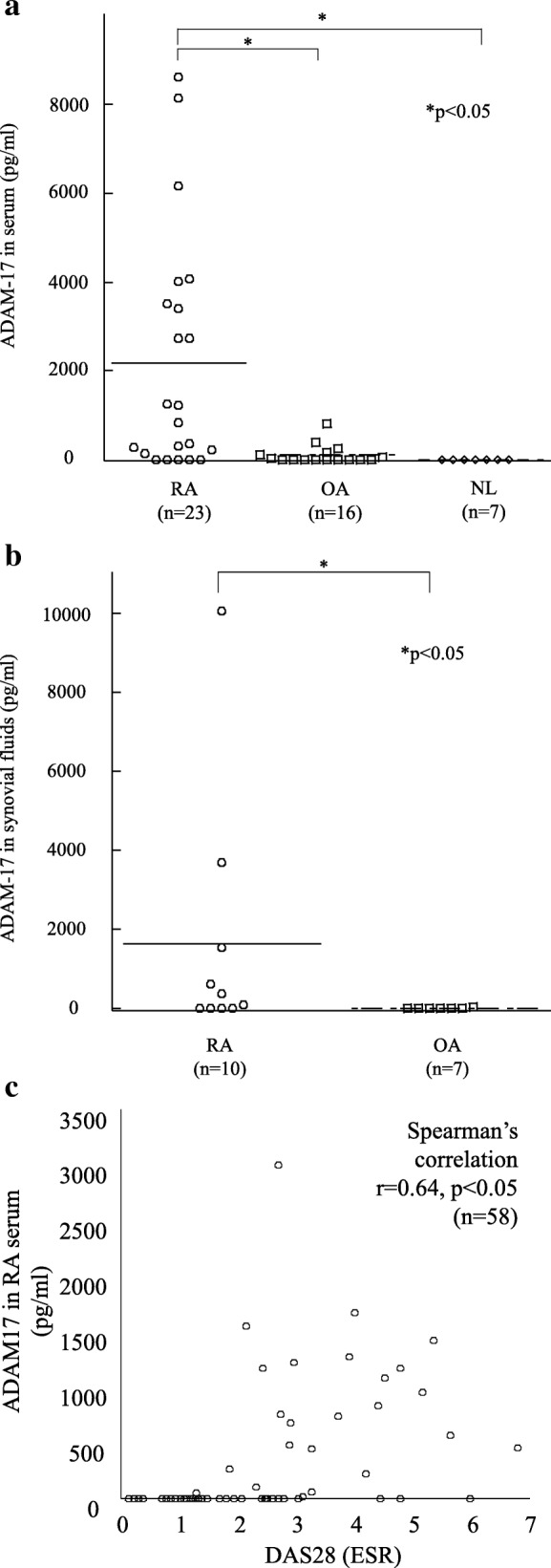
Expression of ADAM-17 in RA serum and SFs. **a** ADAM-17 is higher in RA serum than OA and NL serum. **b** ADAM-17 expression is also higher in RA SFs than OA SFs. **c** The level of ADAM-17 in RA serum is correlated with disease activity. (n = number of patients). *ADAM* a disintegrin and metalloprotease family protein, *DAS28* disease activity score 28, *ESR* erythrocyte sedimentation rate, *NL* normal, *OA* osteoarthritis, *RA* rheumatoid arthritis

Table 1 shows the characteristics of the patients. A total of 78.3% patients were RF positive and 65.2% of patients were ACPA positive. To determine whether ADAM-17 was expressed in serum, we examined ADAM-17 levels in RA, OA, and NL sera using ELISAs. ADAM-17 levels in RA serum (*n* = 23) were significantly higher than those in OA serum (*n* = 16) and NL serum (*n* = 7) (2093 ± 539 pg/ml, 121 ± 53 pg/ml and 0 ± 0 pg/ml, *p* < 0.05, respectively, Fig. 1a). We also examined ADAM-17 levels in RA and OA SFs. ADAM-17 in RA SFs (*n* = 10) was elevated compared to that in OA SFs (n = 7) (1644 ± 952 pg/ml and 4.6 ± 4.3 pg/ml, *p* < 0.05, respectively, Fig. 1b).

**Table 1:** Summary of patient characteristics (mean ± SE)

	RA (*n* = 23)	OA (*n* = 16)	Healthy control (*n* = 7)
Age	56.6 ± 3.4	64.2 ± 6.2	46 ± 4.6
Female, n (%)	17 (74)	13 (81)	3 (43)
Methotrexate dose (mg/week)	7.9 ± 1.2		
Glucocorticoid dose (mg/day)	1.33 ± 0.5		
DAS28 (ESR)	4.81 ± 0.3		
Duration (years)	5.2 ± 1.0		
RF positive (%)	78.3		
ACPA positive (%)	65.2		
Stage (I/II/III/IV)	(7/8/4/4)		
Number of previous biologics	1.0 ± 0.3		

*ACPA* anti-citrullinated protein antibodies, *DAS28* disease activity score 28, *ESR* erythrocyte sedimentation rate, *ICAM-1* intercellular adhesion molecule 1, *RF* rheumatoid factor

To confirm the relationship between ADAM-17 and RA disease activity, we performed correlation analysis with DAS28 erythrocyte sedimentation rate (ESR). We found that ADAM-17 in RA serum showed a significant positive correlation with DAS28 (*r* = 0.64, *p* < 0.05, *n* = 58 patients, Fig. 1c).

These results indicate that the serum level of ADAM-17 is involved in RA disease activity.

### Expression of ADAM-17 in RA STs and FLSs

**Fig. 2:**
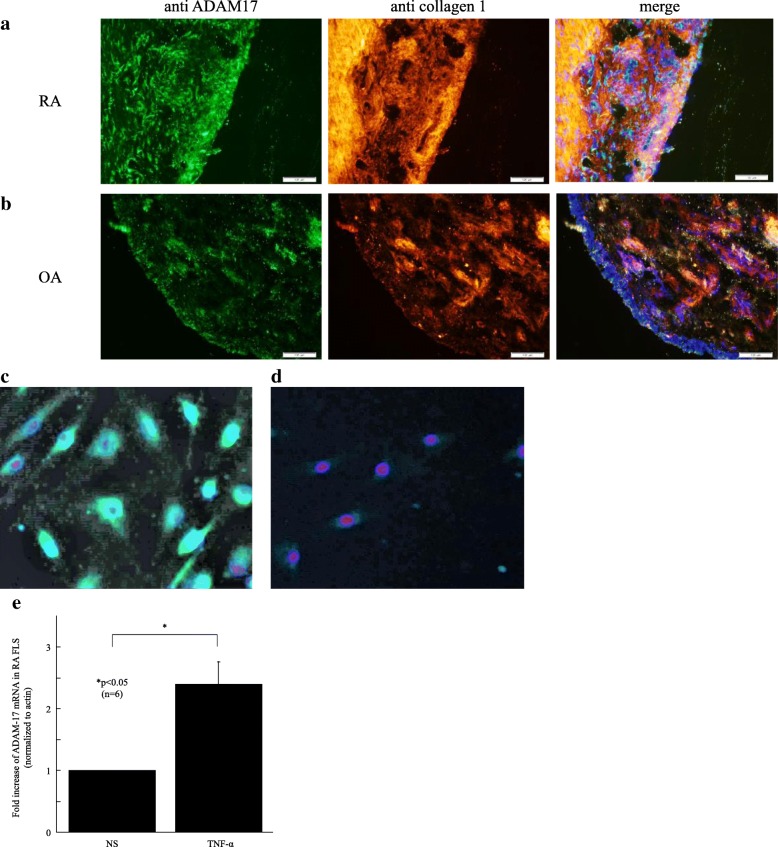
Expression of ADAM-17 in RA STs and FLSs. **a** Representative photomicrographs of synovial tissues (STs) from patient with RA. The *left panel* shows ST staining with rabbit anti-ADAM-17. The *middle panel* shows staining with mouse anti-collagen-1. The *right panel* shows merging of the *left panel* and *middle panel*. The *yellow color* indicates ADAM-17 associated with ST synovium. **b** Representative photomicrographs of ST samples from patient with OA. **c** and **d** Representative photomicrographs of RA fibroblast-like synoviocytes (FLSs). Cryosections and culture cells were stained for ADAM-17 (**c**) or control IgG (**d**). (Original magnification ×200 (**a** and **b**) ×400 (**c** and **d**)) **e** ADAM-17 mRNA in TNF-α stimulated RA FLSs is increased 2.4-fold compared with nonstimulated RA FLSs. *ADAM* a disintegrin and metalloprotease family, *FLSs* fibroblast-like synoviocytes, *OA* osteoarthritis, *RA* rheumatoid arthritis

We then measured expression of ADAM-17 in RA and OA STs using immunofluorescence analyses. We found that ADAM-17-positive cells were expressed on RA cells lining STs (Fig. 2a). However, ADAM-17 was little expressed on OA STs (Fig. 2b). Next, we isolated FLSs from RA STs to assess ADAM-17 expression using immunofluorescence. We demonstrated that ADAM-17 was expressed in RA FLSs (Fig. 2c and d). In addition, we found the expression of ADAM-17 mRNA was induced by stimulation with TNF-α, and elicited a 2.4-fold increase in ADAM-17 levels within 4 h (Fig. 2e). These results indicate that ADAM-17 is involved in RA inflammation.

### The role of proinflammatory mediators in blocking ADAM-17 in RA FLSs

**Fig. 3:**
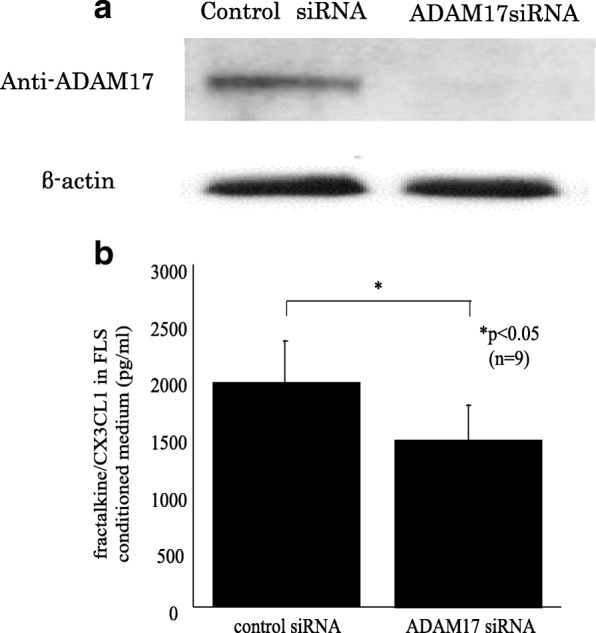
The expression of proinflammatory mediators blocking ADAM-17 in RA FLSs. **a** ADAM-17 siRNA inhibited ADAM-17 expression in RA FLSs. **b** The expression of fractalkine/CX3CL1 was significantly decreased in TNF-α-stimulated ADAM-17 siRNA-transfected RA FLS-conditioned medium compared with TNF-α-stimulated control siRNA-transfected RA FLS-conditioned medium. (n = number of replicates). *ADAM* a disintegrin and metalloprotease family protein, *FLSs* fibroblast-like synoviocytes, *siRNA* small interfering RNA

To confirm the function of ADAM-17 in RA FLSs, we transfected RA FLSs with siRNA against ADAM-17. Specific knockdown of ADAM-17 was confirmed by Western blotting, and the ADAM-17 protein levels in ADAM-17 siRNA-transfected RA FLSs were decreased compared with those in control siRNA-transfected RA FLSs (Fig. 3a).

We examined that the expression of fractalkine/CX3CL1 and VEGF in TNF-α-stimulated RA FLS-conditioned medium because these are important cytokines for RA inflammation and angiogenesis. The expression of fractalkine/CX3CL1 was significantly decreased in TNF-α-stimulated ADAM-17 siRNA-transfected RA FLS-conditioned medium compared with TNF-α-stimulated control siRNA-transfected RA FLS-conditioned medium (Fig. 3b). However, VEGF in TNF-α-stimulated ADAM-17 siRNA-transfected RA FLS-conditioned medium was not changed. These results suggest that ADAM-17 in FLSs is involved proinflammatory cytokine secretion in RA inflammation.

### Blocking ADAM-17 in RA FLSs reduces THP-1 adhesion

**Fig. 4:**
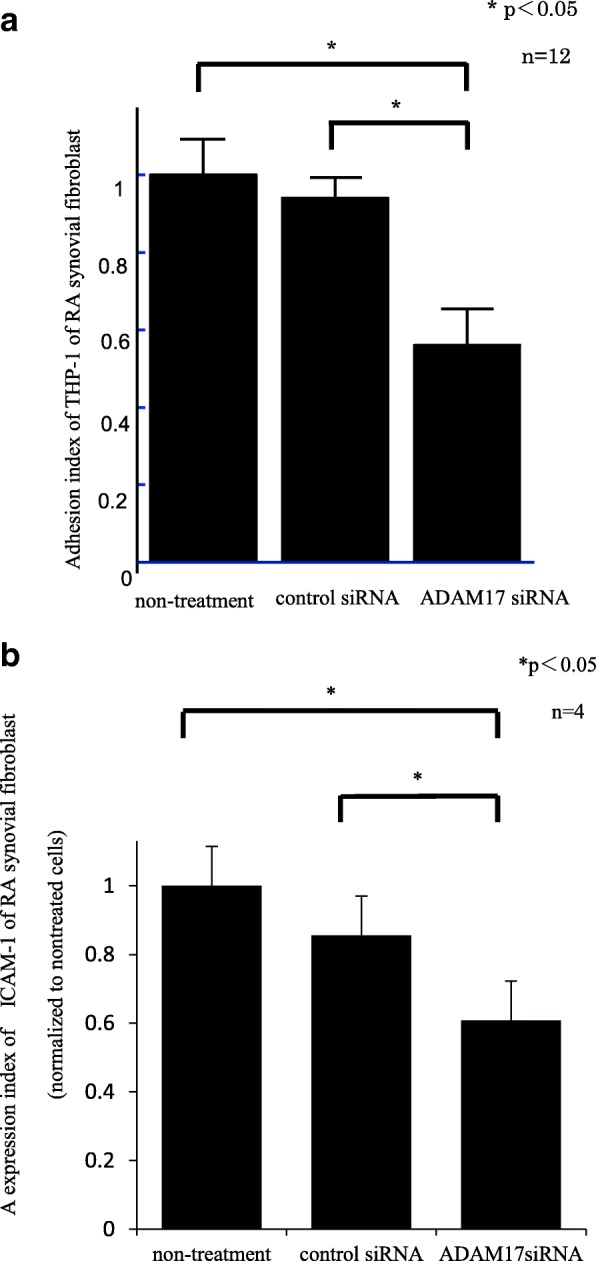
Blocking ADAM-17 in RA FLSs reduces THP-1 adhesion. **a** THP-1 adhesion to ADAM-17 siRNA-transfected RA FLSs was significantly decreased compared with that to control siRNA-transfected RA FLSs. **b** ICAM-1 on TNF-α-stimulated ADAM-17 siRNA-transfected RA FLSs was significantly decreased compared with that on control siRNA-transfected RA FLSs. (n = number of replicates). *ADAM* a disintegrin and metalloprotease family protein, RA rheumatoid arthritis, *siRNA* small interfering RNA

To examine the adhesive function of ADAM-17 in RA FLSs, we used RA FLSs that were transfected with ADAM-17 siRNA or control siRNA. To determine whether ADAM-17 mediates THP-1 adhesion to RA FLSs, we performed in vitro adhesion assays. THP-1 adhesion to ADAM-17 siRNA-transfected RA FLSs was significantly decreased compared with that to control siRNA-transfected RA FLSs (Fig. 4a). In addition, we performed a cell surface ELISA to determine whether cell adhesion molecules were decreased on ADAM-17 siRNA-transfected RA FLSs. ICAM-1, but not VCAM-1, on TNF-α-stimulated ADAM-17 siRNA-transfected RA FLSs was significantly decreased compared with that on control siRNA-transfected RA FLSs (Fig. 4b). These results indicate that ADAM-17 inhibition regulates TNF-α-induced fibroblast adhesion for THP-1 and ICAM-1 expression in RA.

## Discussion

ADAM-17 is believed be associated with inflammation in RA. In this study, we showed that ADAM-17 is expressed on RA STs and FLSs. Charbonneau et al. reported that ADAM-17 was upregulated in both fibroblast- and macrophage-like synovial cells, along with elevated expression of both hypoxia inducible factor-1 and TNF-α in mice [24]. Ohta et al. also reported that ADAM-17 on RA STs was stronger than that on OA STs by immunohistochemistry. In addition, they showed that ADAM-17-expressing cells were mainly CD68+ macrophage-like synovial cells [25]. Our data supported ADAM-17 expression in RA. On the other hand, ADAM-17 was not presented in OA synovial tissues. (This result indicates ADAM-17 might be involved in joint destruction.) We clearly demonstrated that ADAM-17 expression is higher in RA serum than NL serum and is related to disease activity. Taken together, these results indicate that ADAM-17 expression is involved in RA inflammation.

Chemokines are believed to be important in RA inflammation. Fractalkine/CX3CL1 is one of these chemokines and is secreted by ADAM-17 [26]. We found that the expression of fractalkine/CX3CL1 was significantly decreased in TNF-α-stimulated ADAM-17 siRNA-transfected RA FLS-conditioned medium compared with TNF-α-stimulated control siRNA-transfected RA FLS-conditioned medium. Whereas ADAM-17 was expressed on virtually all FLS and was efficiently knocked down to zero/below threshold, only a slight reduction in the expression of fractalkine/CX3CL1 was measured. We previously reported ADAM-10 mediated fractalkine/CX3CL1 and CXCL16 secretion from RA FLS. These results suggest not only ADAM-17 but ADAM-10 regulates membrane-bound chemokines.

Ruth et al. reported that fractalkine/CX3CL1 and its receptor, CX3CR1, were expressed in RA and a rat adjuvant-induced arthritis model, and soluble fractalkine/CX3CL1 was upregulated in RA synovitis [27]. Furthermore, the serum level of fractalkine/CX3CL1 was elevated in patients with RA and was correlated with disease activity [22]. Umemura et al. reported that ADAM17 level was positively correlated with fractalkine/CX3CL1 in RA serum [28]. These results indicate that ADAM-17 in FLSs is involved in RA inflammation by regulating fractalkine/CX3CL1 expression.

ADAM-17 regulates the shedding of a number of transmembrane substrates, including several receptors and/or their ligands, such as VEGF [12]. We showed that VEGF in TNF-α-stimulated ADAM-17 siRNA-transfected RA FLS-conditioned medium was not changed compared with that in TNF-α-stimulated control siRNA-transfected RA FLS-conditioned medium. The VEGF level in conditioned medium from several FLS lines decreased, but the VEGF level in other FLS-conditioned medium increased. These results indicated that VEGF in FLSs could be involved in other proinflammatory mediators or metalloproteases.

We next focused on the influence of ADAM-17 on monocyte adhesion in RA FLSs. We found that THP-1 adhesion to ADAM-17 siRNA-transfected RA FLSs was decreased compared with that to control siRNA-transfected RA FLSs. Additionally, we clearly demonstrated that ICAM-1 on TNF-α-stimulated ADAM-17 siRNA-transfected RA FLSs was decreased compared with that on control siRNA-transfected RA FLSs. ICAM-1 is an adhesion molecule that mediates inflammatory and immune responses and is cleaved on the cell surface by ADAM-17 [29]. On the other hand, ICAM-1 showed only 40% reduction after treatment with ADAM-17 siRNA in RA-FLS. ADAM-17 is one of the cleaving enzymes of ICAM-1 from RA-FLS, and other ADAM family might cleave ICAM-1. This study suggests that ADAM-17 promotes ICAM-1 expression on the cell surface and monocyte adhesion in RA FLSs. However, we found that VCAM-1 on TNF-α-stimulated ADAM-17 siRNA-transfected RA FLSs was not decreased compared with that on control siRNA-transfected RA FLSs. Garton et al. reported that shedding of VCAM-1 is mediated by ADAM-17 in murine endothelial cells [30]. Singh et al. also reported that VCAM-1 was released by ADAM-17 from endothelial cells [31]. In addition, they showed that VCAM-1 ectodomain release was regulated by tissue inhibitor of metalloproteinase-3 in endothelial cells. Meanwhile, we clearly demonstrated that ADAM-17 in FLSs enhances monocyte adhesion via upregulation of ICAM-1, but not VCAM-1, expression. These results indicated that ADAM-17 in FLSs might play a role in inflammation through expression of ICAM-1.

In summary, ADAM-17 is expressed on RA STs and FLSs and is correlated with disease activity in RA. ADAM-17 in FLSs mediates monocyte adhesion and production of proinflammatory cytokines, such as fractalkine/CX3CL1 and ICAM-1. TNF-α activation prior to the assay and thus, ADAM17 might also play an important role in TNF-α mediated cell activation. We next need to demonstrate more functional approach to address the role of ADAM-17 in RA. Taken together, ADAM-17 might be a potential target in inflammatory diseases such as RA.

## Conclusions

Our study demonstrated that ADAM-17 was expressed in RA biological fluids and is correlated with disease activity. ADAM-17 was also expressed in RA synovium and FLS. Blocking expression of ADAM-17 in RA FLS reduced cell adhesion due to inhibition of adhesion and proinflammatory mediator production. We propose that ADAM-17 plays roles in mediating arthritis by this multistep process.

## Abbreviations

ADAM: A disintegrin and metalloprotease family protein
DAB: Chromogen 3,3′-diaminobenzidine
DAPI: 4′,6-diamidino-2-phenylindole
DAS28: Disease activity score 28
ELISA: Enzyme-linked immunosorbent assay
FBS: Fetal bovine serum
FLSs: Fibroblast-like synoviocytes
ICAM-1: Intercellular adhesion molecule 1
IL: Interleukin
NL: Normal
OA: Osteoarthritis
qPCR: Quantitative polymerase chain reaction
RA: Rheumatoid arthritis
SF: Synovial fluid
siRNA: Small interfering RNA
ST: Synovial tissue
TNF-α: Tumor necrosis factor-α
VCAM-1: Vascular cell adhesion molecule 1
